# Magnesium Molybdate: An Efficient Nanosorbent for Methylene Blue Cationic Dye Removal from Aqueous Solutions

**DOI:** 10.3390/molecules30071606

**Published:** 2025-04-03

**Authors:** Ahmed Mohmoud, Souad Rakass, Hicham Oudghiri Hassani, Saheed A. Popoola, Fethi Kooli, Eman Assirey, Mostafa Abboudi

**Affiliations:** 1 Chemistry Department, Faculty of Science, Taibah University, Madinah 30002, Saudi Arabia; caadil77@yahoo.co.uk (A.M.); eman_assirey@hotmail.com (E.A.); abboudi14@hotmail.com (M.A.); 2 Laboratory of Applied Organic Chemistry (LCOA), Chemistry Department, Faculty of Sciences and Techniques, Sidi Mohamed Ben Abdellah University, Imouzzer Road, P.O. Box 2202, Fez 30000, Morocco; 3 Engineering Laboratory of Organometallic, Molecular Materials, and Environment (LIMOME), Chemistry Department, Faculty of Sciences, Sidi Mohamed Ben Abdellah University, P.O. Box 1796, Fez 30000, Morocco; oudghiri_hassani_hicham@yahoo.com; 4 Chemistry Department, Faculty of Science, Islamic University of Madinah, Madinah 42351, Saudi Arabia; abiodun@iu.edu.sa (S.A.P.); fethi_kooli@yahoo.com (F.K.)

**Keywords:** nanosorbent, β-MgMoO_4_, removal, methylene blue, regeneration

## Abstract

The removal of methylene blue (MB) cationic dye from aqueous solutions was investigated by applying magnesium molybdate (β-MgMoO_4_) as a nanosorbent. The β-MgMoO_4_ was synthesized through a simple, rapid, and efficient method. The MB dye removal process was optimized by evaluating various parameters such as temperature, contact time, nanosorbent dosage, pH, and initial cationic dye concentration. The optimal conditions for MB removal were found to be pH 3, with a 99% removal efficiency achieved in just 10 min of contact time, when using an MB cationic dye concentration of 160 ppm. Magnesium molybdate (β-MgMoO_4_) showed a maximum adsorption capacity of 356 mg/g, according to Langmuir model-based calculations. The MB dye removal process occurred spontaneously while being favorable and endothermic. The kinetic investigation showed that the pseudo-second-order model accurately represented the reaction kinetics. The thermal regeneration test results indicated that the removal efficiency remained stable even after three consecutive rounds of reuse. A Fourier Transform Infrared (FTIR) spectroscopic analysis confirmed the adsorption and desorption of MB on β-MgMoO_4_ and its regeneration. Overall, these results indicate that a β-MgMoO_4_ nanosorbent is a favorable and robust adsorbent for the removal of MB cationic dye from wastewater at its maximum capacity.

## 1. Introduction

Manufactured synthetic dyes [1], which are widely used in, but are not limited to, industries such as leather tanning [2], mining [3], electroplating [4], cement [5], chemical fertilizer [6], photoelectrochemical cells [7], textiles [8], cosmetics [9], paper production [10], and coloring agents, for various products, i.e., cotton, food [11], wool, leather, and silk products [12], contain numerous toxic substances [13]. These substances include bases [14], acids [15], dissolved solids, and different colored dyes. Dyes are major sources of water pollution, and, with their complex molecular structures, are highly stable [16], resistant to washing [17], and difficult to degrade [18]. As a result, dyes are considered to be poisonous [19], and they pose a substantial threat to aquatic life, freshwater sources, and human health owing to their toxicity, carcinogenicity, and potential to cause considerable environmental damage [19]. On the other hand, the increasing demand for freshwater, primarily driven by agricultural and industrial activities, has led to global concern regarding the scarcity of this vital resource [20]. To reduce dependency on freshwater sources, using recycled water has been suggested as an alternative solution. This necessitates effective wastewater purification and dye removal methods [21].

In response to this need, various dye removal techniques have been developed, including flocculation, ion exchange, ozone oxidation, chemical coagulation, extraction, membrane separation, biological treatment, electrochemical methods, photodegradation, adsorption, and hypochlorite oxidation [22,23,24,25,26,27,28,29]. Amongst these techniques, adsorption is considered efficient, simple, and low-cost, making it a popular alternative to other expensive treatment procedures [30].

Numerous efforts have been made to develop synthetic or natural materials with high efficiency in removing and degrading the harmful dyes in industrial wastewater using biological, chemical, and physical techniques [31,32].

Activated carbon is universally used as a natural adsorbent as a result of its large surface area [24,25] and high adsorption capacity. However, its use is limited due to its production expenses, regeneration abilities, phase separation difficulties, and low-quality mechanical properties [26]. Thus, research is being conducted to come up with novel adsorbents with high adsorption and regeneration abilities.

Recently, binary metal oxides have attracted great interest because of their prospective performances in various materials [27]. Particularly, metal molybdates (MMoO_4_ or M_2_(MoO_4_)_3_) have drawn attention for their applications in catalysis [33,34], the oxidative dehydrogenation of light alkanes [35,36,37,38,39], and the partial oxidation of hydrocarbons [40], and also for environmental applications such as the photocatalytic oxidation of dyes [41,42,43,44], the oxidation of methylene blue (MB) dye [45], and the removal of phosphate [46].

Magnesium molybdate compounds have drawn great interest for their possible applications as catalysts [47,48,49,50], as phosphors [51,52,53,54,55,56,57,58], as supercapacitors [59], and in lithium-ion batteries [60].

Magnesium molybdate belongs to the wolframite-type phases and exists in two polymorphs: the ß form, which is stable in the temperature range of 500 °C up to 1100 °C, and the cupro scheelite α form, which is generally synthesized using high pressures of about ~5 GPa [14].

So far, different methods of MgMoO_4_ synthesis have been mentioned in the literature, including impregnation synthesis [61], solid-state reaction synthesis [51,52,53,54,55], mechanochemical activation synthesis [62,63], hydrothermal synthesis [56,57,58], coprecipitation synthesis [49,50], an ultrasonic spray pyrolysis method [47], sol–gel synthesis [64], and a citrate method [48]. Different molybdate materials have been explored for the removal of dyes from aqueous solutions, such as nickel molybdate, zinc molybdate, and iron molybdate [43,49,58]. However, until now, the use of ß-MgMoO_4_ for the removal of dyes from aqueous solutions and other environmental applications has not yet been explored.

The current study focused on using ß magnesium molybdate as a cationic dye adsorbent. Several parameters, for instance, temperature, nanosorbent dosage, initial cationic dye concentration, solution pH, and contact time, were all studied to determine their specific effects on MB cationic dye removal activity. An evaluation of adsorption isotherms and kinetics was carried out. Additionally, the removal efficiency, after regenerating the used nanosorbent by calcination at high temperatures, was also investigated.

## 2. Results and Discussion

### 2.1. MB Removal

#### 2.1.1. pH Effect

**Figure 1:**
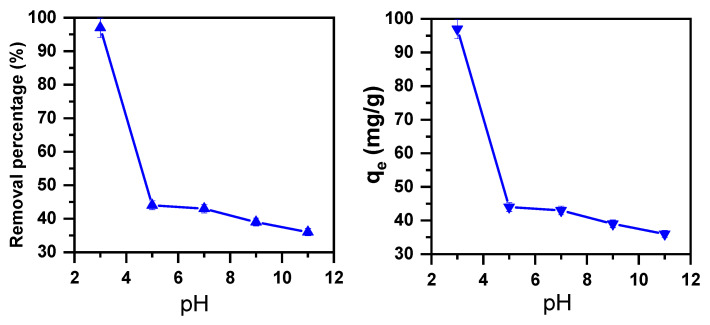
Removal efficiency of β-MgMoO_4_ in a 100 ppm MB cationic solution as function of pH (m_ads_ = 0.1 g; T = 20 °C; t = 30 min).

pH is a necessary parameter in regard to managing dye removal [65]. Therefore, its effect on MB removal using a magnesium molybdate (β-MgMoO_4_) nanosorbent was explored in this study by changing its pH values from 3 to 11 at a stable room temperature (20 °C) with the cationic dye’s initial concentration at 100 ppm. Figure 1 shows that there is a distinctive effect of pH on MB cationic dye removal. For example, a decrease in the MB percentage from 97% to 36% was seen with increasing pH from 3 to 11. A similar trend was observed for the amount of cationic dye removed per unit mass of adsorbent at equilibrium (qe), which decreased from 97 to 36 mg/g. This finding highlights the critical role of acidic conditions.

The presence of excess H^+^ ions is expected to protonate the positive charge on methylene blue dye (MBH_2_^+^), as it is unlikely that these ions would protonate the negative charges on the surface of β-MgMoO_4_. The surface area electrostatic fields of β-MgMoO_4_ and MB were raised by protonation of the surface area charges by excess H^+^ ions. This was mirrored by the higher color removal percentage at lower pH. Thus, the maximum removal of the MB cationic dye using β-MgMoO_4_ nanosorbent was achieved at pH = 3. Similar conclusions were reported by Oudghiri Hassani et al. [66] in a study of an α-zinc molybdate catalyst.

#### 2.1.2. Effect of Adsorbent Dose

**Figure 2:**
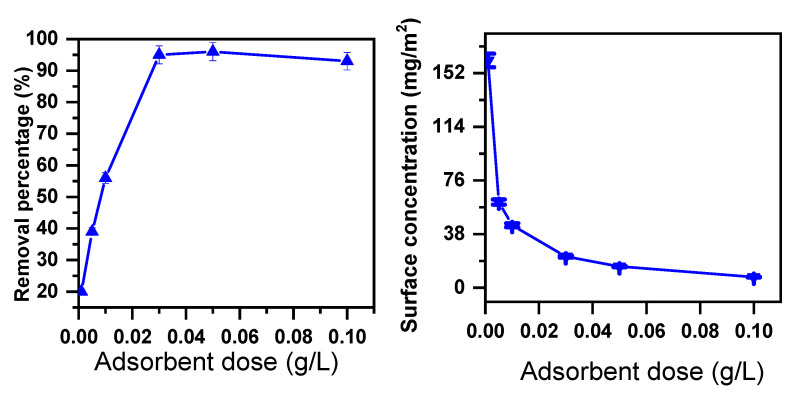
Removal efficacy of β-MgMoO_4_ in 160 ppm MB cationic dye solution as function of adsorption dosage (time = 30 min; temperature = 20 °C).

Adsorbent dosage is considered the most vital parameter in adsorption methods [67,68]. MB dye removal using β-MgMoO_4_, with the initial dye concentration at 160 ppm, was discovered to increase upon altering the dosage of the nanosorbent from 0.0001 to 0.1 g/L. This is described in Figure 2, the percentage (%) of removal of MB cationic dye is increased, while the surface concentration (Γ) (mg/m^2^) decreases if the nanosorbent dosage increases from 0.0001 to 0.1 g/L. This indicates that the available surface area and the adsorption sites were not fully covered by the present MB molecules [69].

#### 2.1.3. Initial Cationic Dye Concentration and Contact Time Effect

Experiments on the effect of adsorption contact time for MB cationic dye solution on β-MgMoO_4_ were conducted at pH = 3 with various controlled contact time intervals varying from 0 min to 120 min. The interaction between the MB cationic dye and the availability of active sites on the β-MgMoO_4_ nanosorbent influences the initial dye concentration.

**Figure 3:**
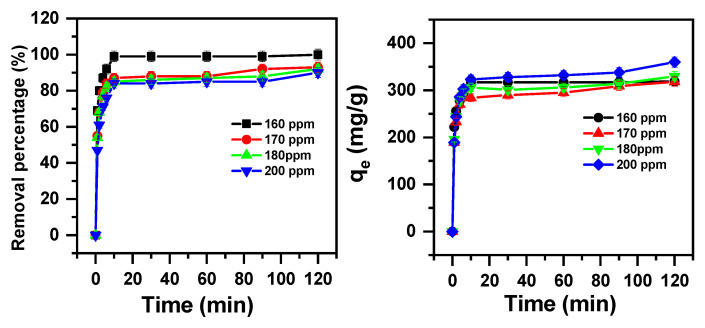
Removal efficacy of β-MgMoO_4_ for MB as a function of initial cationic dye concentration and contact time (pH = 3; m_adsorbent_ = 0.05 g; and T = 20 °C).

The results shown in Figure 3 indicate that the removal of the MB cationic dye by magnesium molybdate attained its highest value of 99% at 10 min, at a concentration of 160 ppm. However, at a concentration greater than 160 ppm, the MB removal percentage reached/decreased to 93%, 92%, and 90% with initial concentrations of 170, 180, and 200 ppm, respectively, at 120 min of contact time.

Furthermore, the actual adsorption capacity results gained at equilibrium increased notably from 317 mg/g to 360 mg/g, with the initial cationic dye concentration rising from 160 mg/L to 200 mg/L. This phenomenon is attributed to the many empty sites on the β-MgMoO_4_ surface. Immediately after the sites of the β-MgMoO_4_ surface adsorption area were fully occupied by MB cationic dye, no more β-MgMoO_4_ surface area sites were available for adsorption; hence, the maximum adsorption capacity was achieved at this stage [70].

In summary, the optimal adsorption contact time for maximum MB removal using β-MgMoO_4_ is 10 min, and the equilibrium adsorption capacity also increases with an increasing initial cationic dye concentration up to a certain limit, after which it starts to decrease.

#### 2.1.4. Temperature Effect

**Figure 4:**
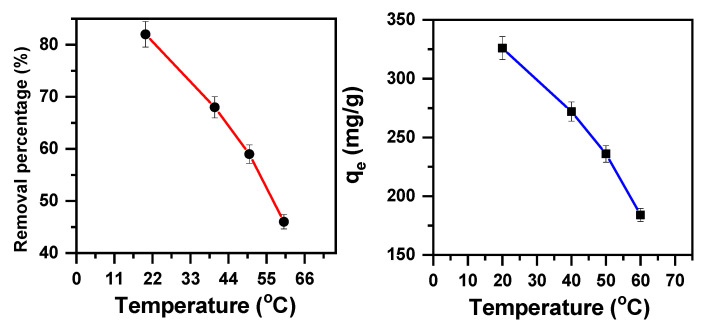
Removal efficiency of β-MgMoO_4_ with a 200 ppm MB cationic solution, as a function of temperature (m = 0.05 g; t = 30 min; and pH = 3).

The experimental process for removing the MB cationic dye was examined at different temperatures, ranging from 20 to 60 °C. The temperature effect results, as confirmed in Figure 4, indicate that the MB removal percentage decreases from 82% to 46% with the initial cationic dye concentration of 200 ppm as the temperature increases. The adsorption capacity also decreases from 326 mg/g to 184 mg/g, demonstrating that exothermic activity controls the adsorption of MB on β-MgMoO_4_.

In summary, the temperature is a key parameter that significantly affects the removal of dyes [71], in this case, using β-MgMoO_4_ as an adsorbent. These results indicate that an exothermic process governs the adsorption of MB onto β-MgMoO_4_, with lower temperatures enhancing removal efficiency.

An important parameter in the adsorption processes is thermodynamic factors [72]. The possibility and the adsorption mechanism are likely to be predicted based on the thermodynamic parameters [73]. These can be calculated via the below-mentioned formulas:

(1) $${\Delta G}^{o}=-RTLnK_{d}$$

(2) $$K_{d}=\frac{C_{a}}{C_{e}}$$

(3) $$LnK_{d}=\frac{{\Delta S}^{o}}{R}-\frac{{\Delta H}^{o}}{RT}$$

K_d_ stands for the distribution constant, T stands for the absolute temperature (K), R stands for the gas constant (J·mol^−1^·K^−1^), ΔG° stands for the free energy, C_a_ stands for the amount of cationic dye adsorbed at equilibrium, C_e_ stands for the equilibrium concentration (mol/L), and ΔS° and ΔH° stand for entropy and enthalpy, respectively.

K_d_ is dimensionless: K_d_ = C_a_/C_e_ in(L/g) ((mg/g)/(mg/L)). To convert it, the obtained value of K_d_, expressed in (L/g), is multiplied by (the molecular weight of the methylene blue (319.85 g/mol) × (the number of moles of water per L of solution (55 moles/L).

**Figure 5:**
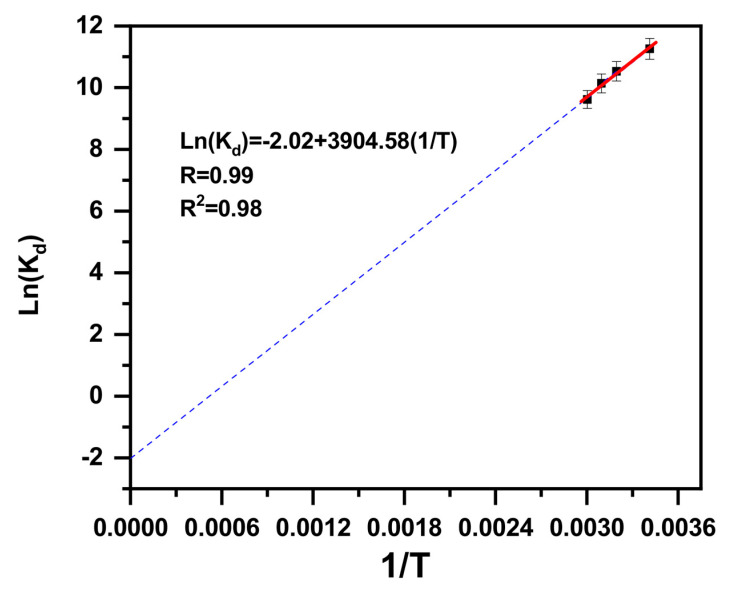
Van’t Hoff plot displaying effects of temperature parameters on MB cationic dye removal by β-MgMoO_4_.

The calculated values of ∆S° and ∆H° were obtained from the intercept, as was the slope of the plot ln K_d_ versus 1/T ($LnK_{d}=-2.09+\frac{3904.58}{T}$ (Figure 5)). The ∆G° values were determined using Formula (1). All calculated data are presented in Table 1.

The negative value of ∆G° indicates a favorable and a spontaneous adsorption process. However, the enthalpy (−32.46 KJ·mole^−1^) and entropy (−0.017 KJ·mole^−1^·K) of the adsorption of MB onto β-MgMoO_4_ are negative, showing the exothermic behavior of the process and a decrease in randomness at the solid–liquid interface during the adsorption process, respectively. The electrostatic attraction between the MB cationic dye and β-MgMoO_4_ (at pH = 3) improves the adsorption forces. Subsequently, the fixation, association, or immobilization of cationic dye molecules on the β-MgMoO_4_ surface area lowers the extent of the freedom of cationic dye molecules. Indeed, based on the ΔG_ and ΔH_ values, it can be postulated that the adsorption of MB is a physical adsorption process [69].

### 2.2. Study of Kinetics

A kinetics test for the removal of MB cationic dye was performed as it provides a hint about the adsorption system [74].

The kinetics data obtained from removing the MB cationic dye by applying the β-MgMoO_4_ nanosorbent were studied with intra-particle diffusion, pseudo-first-order, and pseudo-second-order kinetic models. The equations of the considered models are given in Table 2.

**Figure 6:**
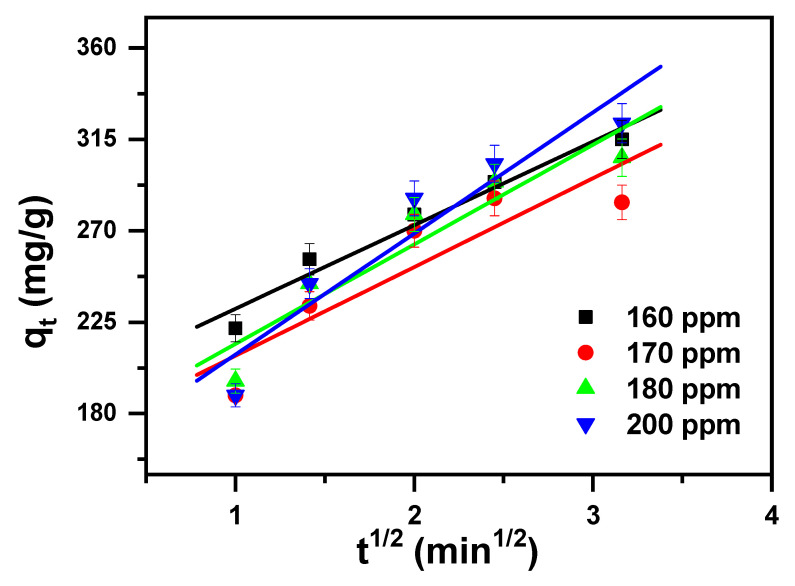
Intra-particle diffusion model plot displaying the effects of contact time and the initial cationic dye concentration of MB removal via β-MgMoO_4_.

**Figure 7:**
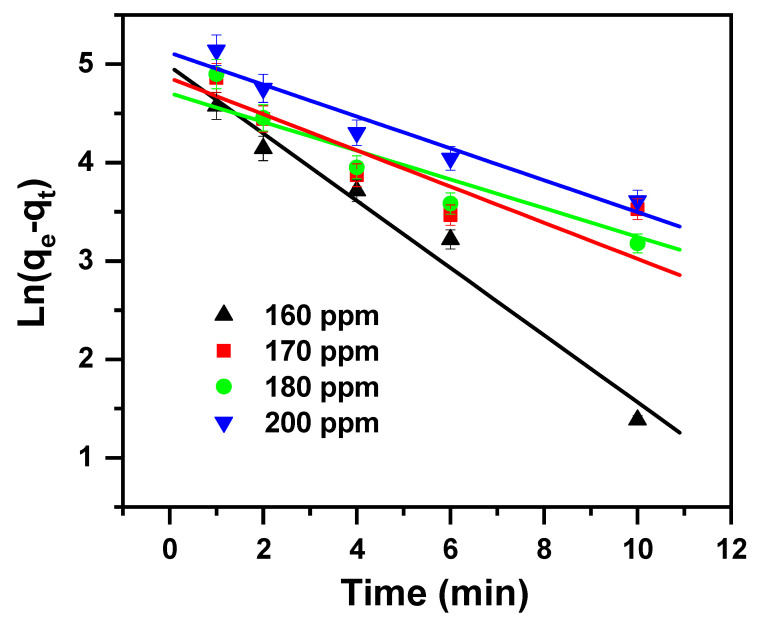
Pseudo-first-order model plot displaying the effects of the initial cationic dye concentration and contact time on MB removal via β-MgMoO_4_.

**Figure 8:**
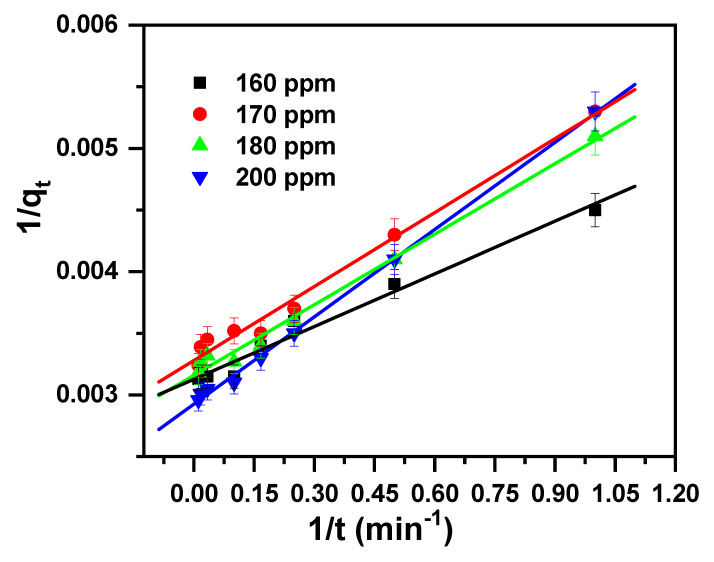
Pseudo-second-order model plot displaying the effects of the initial cationic dye concentration and contact time of MB removal by β-MgMoO_4_.

The kinetic model parameters, intra-particle diffusion, pseudo-first-order and pseudo-second-order are tabulated in Table 3 and shown in Figure 6, Figure 7, and Figure 8, respectively. They vary in terms of the values of their correlation coefficients (R^2^) with their linear regressions. These values were assessed for the examined concentrations and found to be as follows: 0.810 to 0.884 for intra-particle, 0.749 to 0.975 for pseudo-first-order, and 0.979 to 0.998 for pseudo-second-order. As the R^2^ value is equal to or close to 1 for the pseudo-second-order model, it fits perfectly with the investigational data. And hence, the kinetic parameters attained from the pseudo-second-order model agree very well with the parameters from the experimental data. The kinetic studies confirm that the pseudo-second-order model accurately describes the reaction kinetics. This can be seen from the results showing that the pseudo-second-order kinetic model was able to accurately predict the rate of the reaction. This indicates that the reaction mechanism is consistent with the pseudo-second-order kinetic model.

### 2.3. Adsorption Isotherms

It is crucial to study such isotherms due to their perfect explanations when planning adsorption methods [77]. In this current work, four main adsorption models were examined, specifically Dubinin–Radushkevich, Temkin, Freundlich, and Langmuir. These main adsorption models are ruled by the equations given below in Table 4.

**Figure 9:**
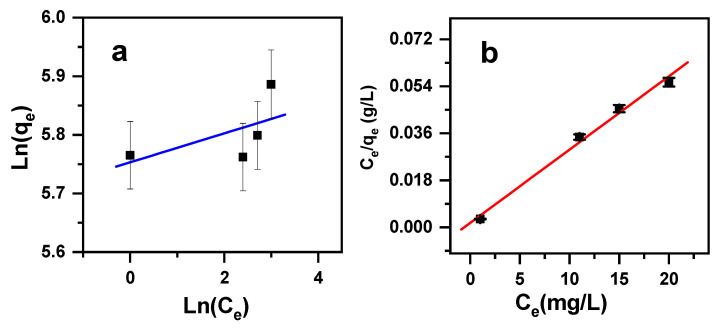
Graphs of (**a**) Freundlich and (**b**) Langmuir isotherms demonstrating initial cationic dye concentration effects on MB removal by β-MgMoO_4_.

The Freundlich, Temkin, Langmuir, and Dubinin–Radushkevich models were tested to see if they perfectly fit the investigational data. The model parameters and the regression correlation coefficients (R^2^) are given in Table 5, as obtained from Figure 9. The maximum value that was obtained for the Langmuir model was R^2^ (0.991), while the Freundlich, Temkin, and D–R models’ fits showed lower values of R^2^ (0.343, 0.338, and 0.210, respectively). Accordingly, Langmuir isotherm fits perfectly with that of the results obtained in the experiments, proposing that the cationic dye removal proceeds via the arrangement of the MB cationic dye monolayer onto the β-MgMoO_4_ nanosorbent surface area, with an adsorption capability of 356 mg/g, which leads to a homogenous surface area. However, the separation factor R_L_, ranging from 0.0031 to 0.0038, indicates favorable dye removal by β-MgMoO_4_. Hence, the explored nanosorbent has excellent removal efficiency in comparison with many other adsorbent materials (Table 6).

### 2.4. Magnesium Molybdate (β-MgMoO_4_) Regeneration and Characterization

#### 2.4.1. Regeneration Efficacy

Various regeneration procedures have been suggested in the literature, such as bio-regeneration, microwave irradiation, thermal treatment, chemical extraction, and supercritical regeneration [83,84,85,86]. In this study, thermal treatment experimental methods were investigated for the regeneration procedure since the chemical structure of the β-MgMoO_4_ removal agent was chemically stable.

**Figure 10:**
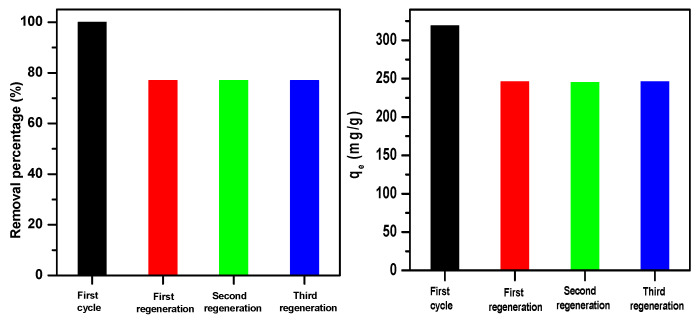
Recycling competences of β-MgMoO_4_ for removal of methylene blue cationic dye (160 ppm, 0.05 g, 30 min).

The results indicate that β-MgMoO_4_ is amenable to regeneration via thermal treatment. Figure 10 demonstrates the recycling efficacy of β-MgMoO_4_ for the removal of methylene blue cationic dye for three consecutive rounds. The results demonstrate that the maximum removal capacity was 246 mg/g even after three cycles of use. It was discovered to be incredibly effective in regenerating β-MgMoO_4_ adsorbent by calcination at 400 °C in an air atmosphere, demonstrating its exceptional reusability.

In summary, the thermal treatment method is effective in regenerating β-MgMoO_4_ adsorbent, and these results suggest that it has excellent reusability for the removal of MB cationic dye from aqueous solutions. These findings are promising for the practical application of β-MgMoO_4_ as an adsorbent for the removal of MB cationic dye from wastewater.

#### 2.4.2. FT-IR Spectroscopy

**Figure 11:**
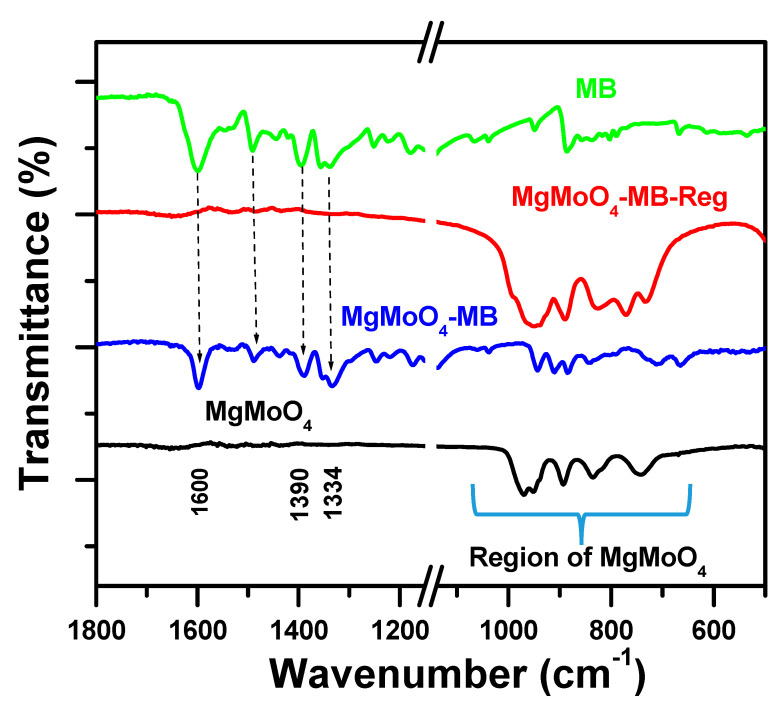
FT-IR spectra of MgMoO_4_, MgMoO_4_-MB, MgMoO_4_-MB-Reg, and MB.

The spectra of β-MgMoO_4_ before and after exposure to MB dye were compared, as displayed in Figure 11. The FT-IR spectra show clear flexing and stretching vibrational characteristics of the metal–oxygen bonds located at frequencies lower than 1100 cm^−1^, corresponding to beta magnesium molybdate [79]. The spectrum of pure MB cationic dye revealed bands between 1700 and 1000 cm^−1^ [79]. After MB removal, further bands located at 1600 cm^−1^ were observed in the FT-IR spectrum of (MgMoO_4_-MB), which are credited to MB C=C bond stretching, indicating the existence of MB due to their attachment to the β-MgMoO_4_ active sites.

The FTIR spectrum of regenerated MgMoO_4_ (MgMoO_4_-MB-Reg) after thermal treatment was comparable to that of fresh MgMoO_4_, indicating that the attached MB cationic dye molecules on the surface completely combusted. The resulting spectrum confirms the cleanliness of the regenerated adsorbent and its reusability.

### 2.5. MB Removal Mechanism

The data showed that there was no evidence of any intermediate molecules or any chemical breaking down of MB cationic dye during its removal following the adsorption process.

**Figure 12:**
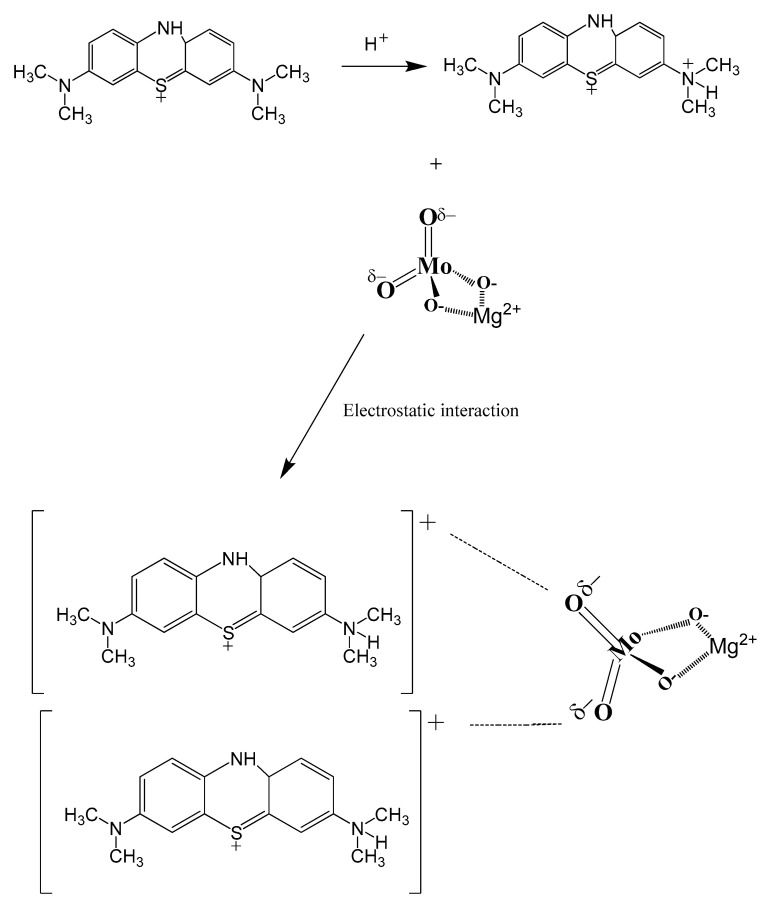
Schematic mechanism of MB cationic dye removal employing magnesium molybdate nanosorbent.

The efficacy of MB cationic dye removal using β-MgMoO_4_ nanoparticles was found to decrease with an increase in pH values up to 11, which could be due to the acidic media. Building on this, we can propose a mechanism by which the protonation of MB cationic dye in acidic media leads to positively charged ammonium units (–N^+^) [66]. The oxygen atoms of the magnesium molybdate nanoparticles and these ammonium components engage electrostatically in the following step, enabling the adsorption of MB cationic dye on the catalytic surface. This mechanism is depicted in Figure 12.

In summary, the proposed mechanism suggests that the removal of MB by β-MgMoO_4_ nanoparticles occurs through an electrostatic interaction involving the nanoparticles’ surface and the positively charged ammonium entities of the protonated MB. These results suggest that β-MgMoO_4_ nanoparticles are a promising adsorbent for removing cationic dyes from wastewater.

The SEM images clearly illustrate the structural changes in β-MgMoO_4_ before and after the adsorption of MB cationic dye, as well as after the regeneration and reuse of the nanosorbent. The highly porous structure of the starting material allows for better adsorption of the dye, while the filled pores in the sample after adsorption indicate that the active sites on the surface were filled by MB cationic dye molecules. The agglomeration of particles after regeneration explains the decrease in dye removal efficiency, as some of the active sites may have been blocked by agglomerated particles. However, the morphology of β-MgMoO_4_ remains stable and unchanged even after multiple reuses and regeneration, indicating that it is a reliable and reusable nanosorbent for removing MB cationic dye from aqueous solutions.

**Figure 13:**
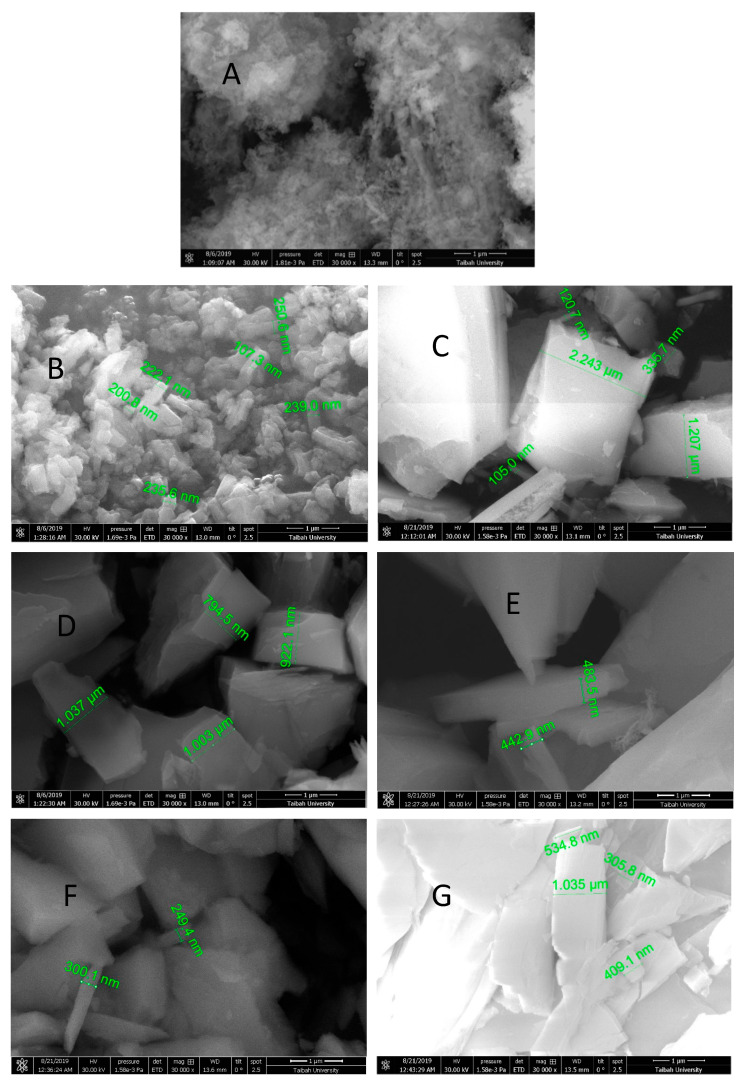
SEM micrographs of (**A**) pure magnesium molybdate (β-MgMoO_4_), (**B**) the morphology of β-MgMoO_4_ after MB dye removal, (**C**) the morphology of β-MgMoO_4_ after the first regeneration, (**D**) the morphology of β-MgMoO_4_ after the second removal cycle of MB cationic dye, (**E**) the morphology of β-MgMoO_4_ after the second regeneration process, (**F**) the morphology of β-MgMoO_4_ after the third removal cycle of MB cationic dye, and (**G**) the morphology of β-MgMoO_4_ after the third regeneration process.

Note that in all of the cases, the particles were of nanoscale size, and the size values obtained with different techniques were of the same order of magnitude. However, the value of approximately 150–200 nm for particles assembled in agglomerates of about 1–2 microns obtained using the SEM technique was much higher than that of 36 nm obtained using XRD. The smaller value from the XRD analysis can be explained by the fact that XRD determines the crystallite size and not the particle size. Agglomeration of the crystallites results in the formation of particles of greater size. As a result, there will be less adsorption of MB molecules in the case of an agglomerated powder (78% after regeneration). In XRD, the crystallite size is measured even if it is agglomerated into larger particles because the crystallites are separately crystallized (Figure 13).

## 3. Experimental Materials and Methods

### 3.1. Magnesium Molybdate Preparation

The chemicals, oxalic acid H_2_C_2_O_4_,2H_2_O, magnesium nitrate Mg(NO_3_)_2_,6H_2_O, and ammonium molybdate (NH_4_)_6_Mo_7_O_24_,4H_2_O, were acquired from Sigma-Aldrich (St. Louis, MO, USA) and consumed in their original states without any modifications, except for the MB dye, which was provided by Panreac (Barcelona, Spain). The magnesium molybdate nanosorbent was synthesized using a new method, the solid-state reaction synthesis method, from the magnesium molybdenum complex [50]. This new method is very simple to conduct; the preparation is carried out without the use of any solvents in two steps and a short time. The process involved mixing and grinding together magnesium nitrate, ammonium molybdate, and oxalic acid in their solid state in a ratio of 1/7:3:1, respectively. The resulting milled combination was then heated at 160 °C on a hotplate, and then burned inside a tubular furnace at a temperature of 500 °C under static air and kept for two continuous hours.

### 3.2. Experimental Adsorptions

Various concentrations of solutions were prepared from a stock solution, and the removal of MB cationic dye was performed by continuously stirring a specific amount of adsorbent into a 100 mL MB cationic dye solution with specified concentrations, at various temperatures, and with different contact times. The required pH of the testing solution was controlled by adjusting with a base (0.1N NaOH) and/or an acid (0.1N HCl). Towards the end of fixed time intervals, the solution was filtered and examined with a UV–visible spectrometer at λ_max_ = 665 nm. The percentages of MB removed, the surface concentration, and the quantity adsorbed, at equilibrium, were calculated using specific formulas.

$$Removal\%=\frac{Co-Ce}{Co}\times 100$$

$$\Gamma =\frac{Co-Ce}{M\cdot a_{s}}\times V$$

$$qe=\frac{Co-Ce}{M}\times V$$

where C_0_ is the initial concentration of the MB cationic dye in solution, Ce is the equilibrium concentration of the MB cationic dye, M is the mass of the used adsorbent, $a_{s}$ is the specific surface area, and V is the measured solution volume.

The results were obtained successively in triplicate, and the % uncertainty was found to be less than 3%, indicating that the results are precise and reliable.

### 3.3. Adsorbent Regeneration Procedure

A fixed concentration of 160 ppm at pH = 3 was used for the generation experiments. The fresh β-MgMoO_4_ was dried after filtration at 100 °C and then calcined at 400 °C for 1 h under atmospheric air. Then, the calcined β-MgMoO_4_ was checked for recycling potential under the same conditions as the fresh β-MgMoO_4_. The recycling process was repeated for three successive rounds under the same settings. The concentration of the removed MB in the solution was analyzed using a UV-Vis spectrophotometer analytical instrument, and the MB removal percentage as well as the amount removed at equilibrium were calculated using the formulas mentioned earlier.

### 3.4. Characterization

**Figure 14:**
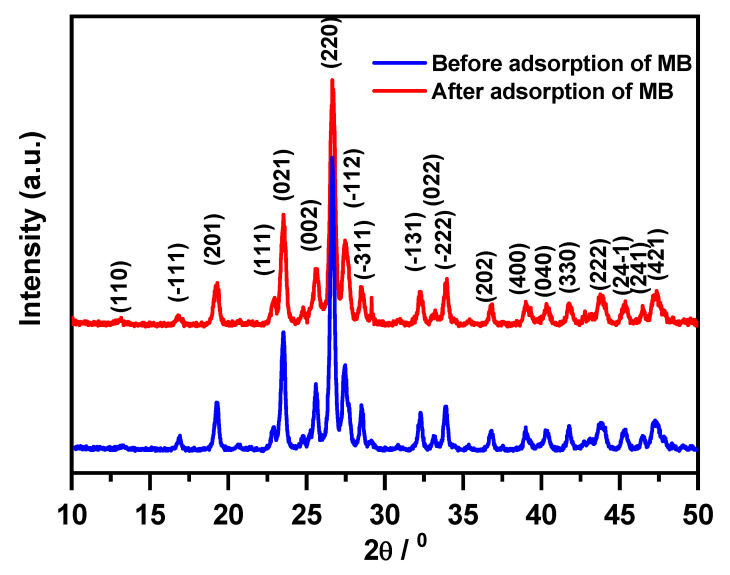
X-ray diffraction pattern of the prepared MgMoO_4_ nanoparticle powder before and after adsorption of MB.

The phase composition of the synthesized nanosorbent before and after its use for the removal of MB dye was analyzed by the XRD technique (X-ray diffractometer 6000, Shimadzu, Tokyo, Japan, equipped with λCu-Kα = 1.5406 Ǻ and Ni filter). The XRD pattern exhibited the characteristic reflections of pure β-MgMoO_4_ phase, and they were indexed based on J.C.P.D.S card number 72-2153 (Figure 14). The Scherrer equation was used to estimate the particle size from the XRD pattern of the as-prepared nanoparticles as follows: D_XRD_ = 0.9λ/(βcos θ); here, D_XRD_ is the average particle diameter, λ is the Cu kα wavelength, β is the full-width at half-maximum (FWHM) of the diffraction peak, and θ is the diffraction angle. The intense peak at 2 θ = 26.64^o^ (220), which correlates to the highest d spacing, was picked to determine the crystallite size D_XRD_, which was discovered to be 36 nm for both cases. A Micromeritics ASAP 2020 surface area and porosity analyzer, (Micromeritics, Norcross, GA, USA), was used to measure the adsorption–desorption isotherms.

The specific surface area of the magnesium molybdate MgMoO_4_ prepared was estimated by the Brunauer–Emmett–Teller technique (BET). It was found to be SBET = 20.3 m^2^/g. FTIR spectroscopy was performed using KBr pellets and IR Affinity 1S Shimadzu equipment (Shimadzu, Tokyo, Japan) within the range of 400 to 4000 cm^−1^ to identify the presence of the methylene blue cationic dye on β-MgMoO_4_ nanosorbent following the experimental adsorption and desorption tests.

SEM studies were carried out using a Quanta Feg-250 Thermo Fisher Scientific instrument (Thermo Fisher Scientific, Hillsboro, OR, USA) to obtain micrograph images and identify the adsorption–desorption steps of the methylene blue cationic dye on the β-MgMoO_4_ nanosorbent. UV–visible spectrophotometer studies were performed using a Thermo Scientific Genesys 10S instrument (Thermo Fisher Scientific, Madison, WI, USA) to determine the % removal of MB and the concentration at equilibrium.

## 4. Conclusions

β-MgMoO_4_ was successfully synthesized and investigated as an MB nanosorbent in aqueous solution. It demonstrated powerful pH dependence with an accomplished removal efficiency of 99% after only 10 min of contact time, with an initial 160 ppm dye concentration of pH = 3 employed. Additionally, the kinetic investigations demonstrated that MB removal conformed to a pseudo-second-order model. Thermodynamics suggested that the Langmuir isotherm was the most appropriate model in terms of its fit with the experimental adsorption data. Remarkably, calculations of the Langmuir model displayed that the removal amount reached a maximum of 356 mg/g. Moreover, β-MgMoO_4_ was effectively regenerated with success after calcination at 400 °C and could be reused multiple times without a significant reduction in efficiency. Remarkably, when β-MgMoO_4_ was tested, it still exhibited excellent removal performance for the studied MB dye, even after three consecutive rounds of reuse, while retaining high removal efficiency. Overall, the data presented that β-MgMoO_4_ is indeed an effective nanosorbent that is capable of outstanding removal performance and provided consistent results over multiple recycling tests.

## Figures and Tables

**Table 1:** MB removal by β-MgMoO_4_: thermodynamic parameters.

Adsorbent	Adsorbate	∆H° (KJ·mol^−1^)	∆S° (KJ·mol^−1^·K)	∆G° (KJ·mol^−1^)			
β-MgMoO_4_	MB	−32.463	−0.017	293 K	313	323 K	333 K
				−27.425	−25.649	−24.699	−23.422

**Table 2:** Kinetic models’ equations.

Model	Equation	Parameters
Pseudo-first-order (PFD) [58].	$Ln(q_{e}-q_{t})=Lnq_{e}+K_{1}t$ (4)	q_t_ stands for the removal capacity at time t (mg/g); q_e_ stands for the removal capacity at equilibrium (mg/g); K_1_ stands for the rate constant of pseudo-first-order adsorption (1/min).
Pseudo-second-order (PSD) [75].	$\frac{1}{q_{t}}=(\frac{1}{K_{2}q_{e}^{2}})\frac{1}{t}+\frac{t}{q_{e}}$ (The plot of 1/qt vs.1/t) (5)	q_t_ stands for the removal capacity at time t (mg/g); q_e_ stands for the removal capacity at equilibrium (mg/g); K_2_ stands for the pseudo-second-order rate constant (g·mg^−1^·min^−1^).
Intra-particle diffusion (IPD) [76].	$q_{t}=K_{I}t^{0.5}+l$ (6)	I (mg/g) and K_I_ (mg/(g·min^0.5^)) are the intra-particle diffusion constants; q_t_ stands for the removal capacity (mg/g) at time t; t stands for the contact time (min).

**Table 3:** MB cationic dye removal by β-MgMoO_4_: kinetic parameters.

Dye C_i_mg/L	Pseudo-First-Order				Pseudo-Second-Order			Intra-Particle Diffusion Model		
	q_exp_ (mg/g)	q_e_ (mg/g)	k_1_ (1/min)	R_1_^2^	q_e_ (mg/g)	k_2_ (g/mg min)	R_2_^2^	I (mg/g)	k_i_ (mg/g min^0.5^)	R_3_^2^
160	319	145	0.341	0.975	319	2.20	0.979	190	41.17	0.958
170	318	110	0.146	0.749	305	1.64	0.987	165	43.71	0.810
180	330	129	0.184	0.927	316	1.65	0.990	165	49.04	0.884
200	360	167	0.162	0.939	341	1.24	0.998	149	59.63	0.904

**Table 4:** Adsorption isotherm models for MB cationic dye removal via β-MgMoO_4_.

Model	Equation	Parameters
Freundlich [78].	$Lnq_{e}=Lnq_{F}+\frac{1}{n}LnC_{e}$ (7)	q_F_ stands for the Freundlich constant (mg^(1−1/n)^L^1/n^g^−1^), n: stands for the heterogeneity factor (g/L); q_e_ stands for the amount of MB cationic dye adsorbed by β-MgMoO_4_ at equilibrium (mg/g); C_e_ stands for MB the cationic dye concentration at equilibrium (ppm).
Langmuir [78].	$\frac{C_{e}}{q_{e}}=\frac{1}{q_{m}K_{L}}+\frac{C_{e}}{q_{m}}$ (8)	q_e_ stands for the amount of MB cationic dye adsorbed by β-MgMoO_4_ at equilibrium (mg/g); C_e_ stands for the MB cationic dye concentration at equilibrium (ppm); q_m_ stands for the highest amount of MB cationic dye removed by β-MgMoO_4_ (mg/g); K_L_ stands for the Langmuir adsorption constant (L/mg).
	$R_{L}=\frac{1}{1+K_{L}C_{i}}$ (9)	C_i_ stands for the initial cationic MB dye concentration; K_L_ stands for the Langmuir constant; R_L_ stands for the values that indicate that the removal of MB cationic dye may be either linear (R_L_ = 1), irreversible (R_L_ = 0), favorable (0 < R_L_< 1), or unfavorable (R_L_ > 1).
Dubinin–Radushkevich (D-R) [79].	$Lnq_{e}=Lnq_{m}-K\varepsilon^{2}\;$(10) $\varepsilon =RTLn(1+\frac{1}{C_{e}})$ (11)	K stands for the sorption energy constant (mol^2^/kJ^2^); ε stands for the Polanyi potential; T stands for the temperature (K); R stands for the universal gas constant (8.314 J·mol^−1^ K^−1^); q_m_ stands for the theoretical saturation capacity; C_e_ stands for MB cationic dye concentration at equilibrium (ppm).
Temkin [80].	$q_{e}=B_{T}LnA_{T}+B_{T}LnC_{e}$ (12)	b_T_ stands for the Temkin constant associated with the heat of sorption (J/mol); B_T_ = R_T_/b_T_; R stands for the gas constant (8.314 J/mol K); A_T_ stands for the Temkin isotherm constant (L/g); T stands for the absolute temperature (K).

**Table 5:** MB removal by β-MgMoO_4_: isotherm parameters.

Langmuir				Freundlich			Temkin			Dubinin–Radushkevich		
q_m_ (mg/g)	K_L_ (L/mg)	R^2^	Range R_L_	q_F_ (mg^(1−1/n)^L^1/n^g^−1^)	1/n	R^2^	A_T_ (L/g)	B_T_	R^2^	q_m_ (mg/g)	R^2^	E (Kj/mol)
356	1.63	0.991	0.0031–0.0038	315	0.025	0.343	3 × 10^16^	8.306	0.338	343	0.210	12.8

**Table 6:** Maximum adsorption capacity (q_m_) of different nanosorbents for the removal of MB cationic dye as reported previously in the literature.

Nanosorbent	q_m_ (mg/g)	Reference
Magnetic iron oxide nanosorbent	25.54	[26]
Fe_3_O_4_ magnetic nanoparticles modified with 3-glycidoxypropyltrimethoxysilane and glycine	158.00	[81]
Zinc molybdate nanoparticles	217.86	[66]
Calcined titanate nanotubes	133.33	[82]
Molybdenum trioxide nanorods and stacked nanoplates	152.00	[83]
Magnesium molybdate (β-MgMoO_4_)	356.00	This current work

## Data Availability

The data presented in this study and the samples of the compounds are available upon request from the corresponding author.
